# Lower trapezius myocutaneous flap repairs adjacent deep electrical burn wounds

**DOI:** 10.1186/s40001-020-00465-8

**Published:** 2020-12-01

**Authors:** Chengying Meng, Yuyao Liu, Huan Wang, Youjun Sun, Shiping Lu, Yan Zhou, Jiayan Hu, Youxin Yu, Linsen Fang, Yexiang Sun, Delin Hu

**Affiliations:** 1 Department of Burn, The First Affiliated Hospital of Anhui Medical University, No. 218 Jixi Road, Hefei, Anhui 230022 People’s Republic of China; 2 Operating Room of Burn Department, The First Affiliated Hospital of Anhui Medical University, No. 218 Jixi Road, Hefei, Anhui 230022 People’s Republic of China

**Keywords:** Electrical burns, Neck, Shoulder and back, Lower trapezius muscle flap

## Abstract

**Background:**

Local tissue damage caused by electrical burns is often deep and severe. High-voltage electrical burns are common in the head, neck and torso areas. These are mostly caused by direct contact with the power supply and are often accompanied by deep injuries of the nerve, blood vessel, muscle, tendon, and bone. We must pay great attention to the clinical treatment of these parts injured by electrical burn.

**Case presentation:**

The first case involved a migrant worker who touched a 6-kV high-tension wire when welding steel; this electric shock caused burns in many places. Deep electrical burn wounds were mainly located on the left shoulder and back, characterized by widespread skin and soft tissue defect and bone necrosis. We utilized a lower trapezius myocutaneous flap to repair these wounds in the neck and back caused by deep electrical burns. The flap survived completely and the wound was effectively repaired. The function and shape of the shoulder and back after the restoration were satisfactory. The second case involved a 29-year-old who accidentally touched a high-voltage wire while working and was burned by a 30,000-V electric shock. His wounds were mainly located on the left head, neck, back and left upper limbs. We designed a 30 cm × 12 cm right trapezius myocutaneous flap which completely covered the wound surface; the electrical burn wounds on the neck and back were effectively repaired. After the electrical burn wound was repaired, the neck function returned to normal with a satisfactory shape.

**Conclusion:**

The authors report two cases of patients who were burned by high voltage. We used lower trapezius myocutaneous flaps to repair their wounds, which achieved satisfactory clinical results. This study has provided a reliable surgical method for the clinical treatment of deep electrical burn wounds in the neck, shoulders and back.

## Background

Local tissue damage caused by electrical burns is often deep and severe. After debridement, tendons, nerves, important blood vessels, bones, and joints are easily exposed, making the wound difficult to repair [1]. In the past, these required multiple surgical treatments, which were of high risk and long duration; this could have a serious impact on the patient's shape and function [2]. Deep electrical burns on the neck, shoulders and back are complicated by their complex anatomical structure which is adjacent to vital organs. The long exposure time of the wound predisposes it to serious complications such as major bleeding, making it more difficult to clinically repair the wound. The trapezius myocutaneous flap is a pedicled composite tissue flap consisting of skin and soft tissue on the surface of the shoulders, neck and trapezius. It can repair tissue defects on the neck, cheeks, skull, mouth, and even the skull base. The trapezius muscle is a flat muscle located on the back, near the head and neck. Beneath the trapezius muscle are the levator scapulae, the large rhomboid muscles and the scapula. The texture of the skin is similar to that of the head and neck. It is commonly used for the repair and reconstruction of various defects in the site [3]. According to the function of the trapezius and direction of its muscle fibers, it can be divided into upper, middle, and lower parts [4]. Multiple types of myocutaneous flaps can be designed from the trapezius, such as a lateral trapezius myocutaneous flap, upper trapezius myocutaneous flap, lower trapezius myocutaneous flap, and scapular skeletal myocutaneous flap. Clinical trials have been carried out based on a large number of anatomical studies, especially in the reconstruction of the back, head and neck defects, and in large-area defects after surgery and repair of bone defect. Meanwhile, there are also ongoing studies and applications of myocutaneous flaps, fasciocutaneous flaps, and skeletal myocutaneous flaps based on the trapezius muscle. We used lower trapezius myocutaneous flaps to repair the wounds of two patients with deep electrical burns on the shoulder, neck, and back. These have achieved satisfactory clinical results. According to our research, we found that the lower trapezius myocutaneous flap is convenient, feasible and effective in repairing neck, shoulder, and back defects.

## Case presentation

### Case 1

**Fig. 1:**
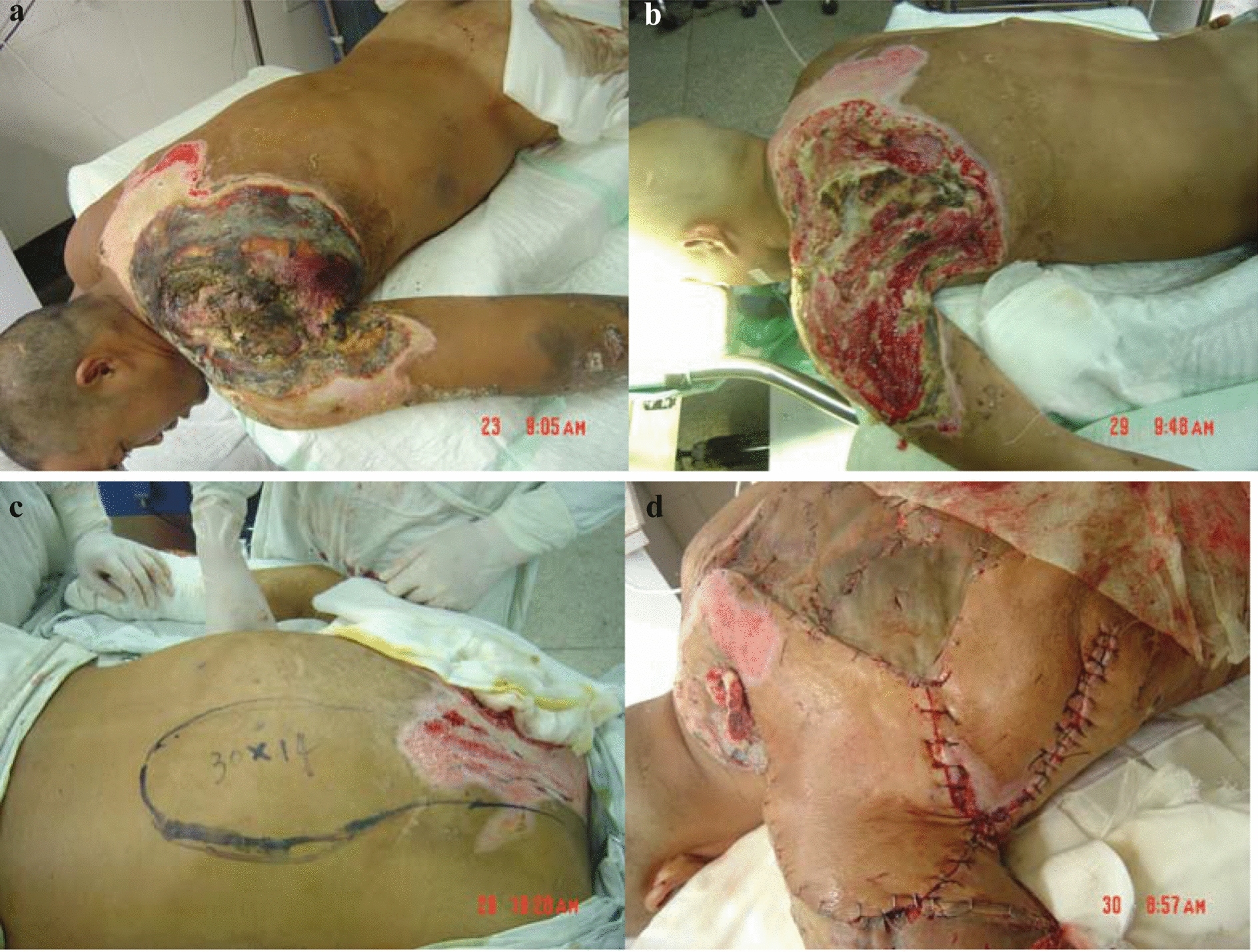
**a** Preoperative, **b** after the first debridement, **c** myocutaneous flap design, **d** myocutaneous flap coverage

**Fig. 2:**
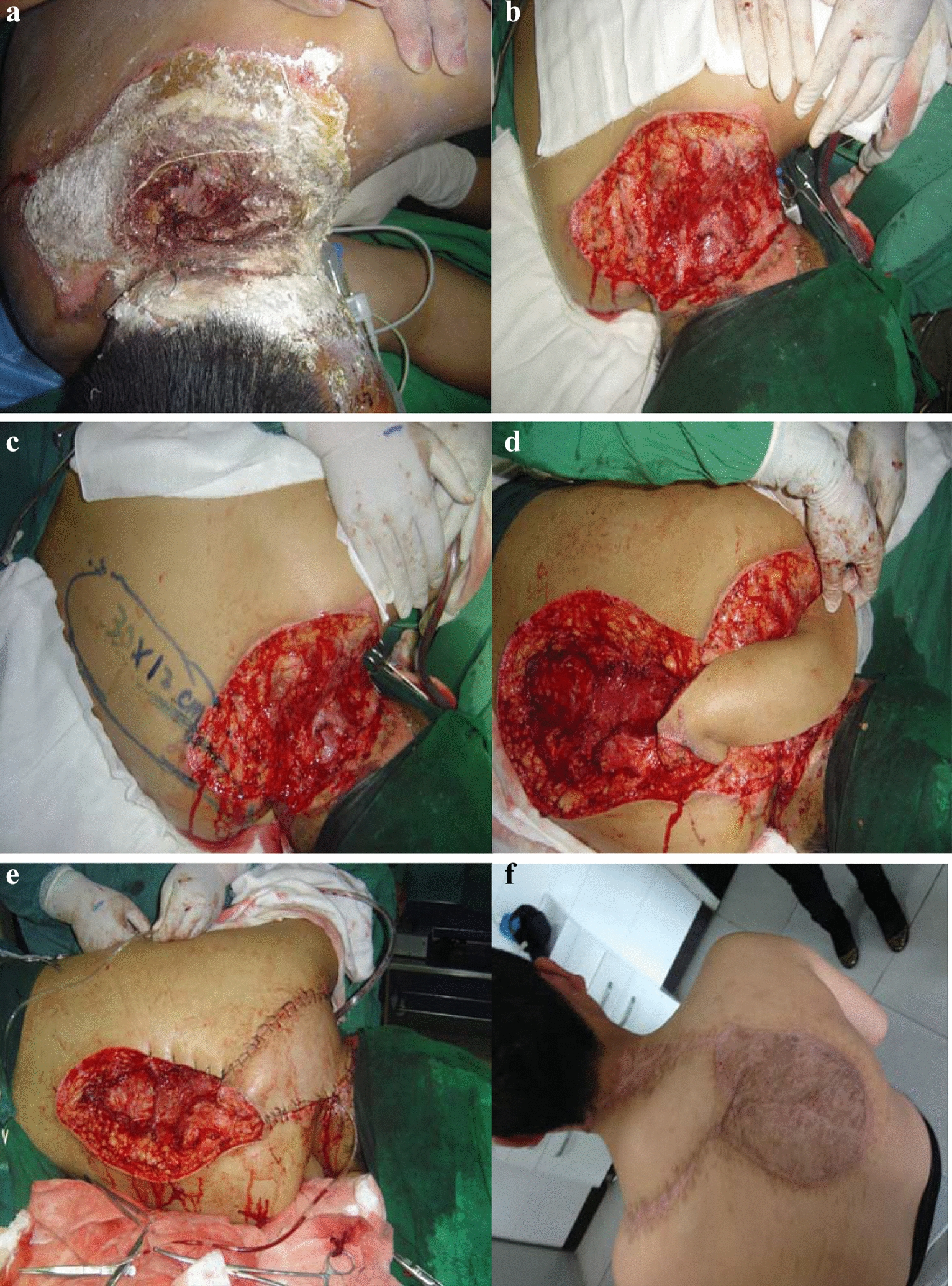
**a** The wound before operation, **b** after the wound was debrided, **c** flap design, **d** shear flap, **e** flap was transferred to repair the wound, **f** the patient's condition after half a year

The patient is a 31-year-old male worker who touched a 6-kV high-tension wire while welding steel, and was badly burned by electricity. The patient woke up after about 10 min in coma. The deep electrical burn wounds were mainly located on the left shoulder and back (Fig. 1a), characterized with large areas of skin, soft tissue, and bone damage. He was treated in a local hospital for 8 days. No electric surgery was performed on the electrical burn wounds. He was then transferred to our department with a diagnosis of high voltage electric injury 40% II–III° in the trunk, upper left limb, lower limb, and buttock with infection. When he was admitted to our hospital, his general condition was poor, with a temperature of 39.2 °C, respiratory rate of 24 breaths/min, pulse of 100 beats/min, and normal blood pressure. After routine examinations, we assessed that the patient’s visceral function was normal and his internal environment was stable. The wound was dressed by sulfadiazine silver paste and exposed. The patient was given anti-infection and symptomatic supportive care. On the 6th day of admission, the wound on the left shoulder and back was debrided and dissected. In the meantime, xenobiotic skin (alcohol chlorhexidine pigskin) dressing was performed. The wounds on the left shoulder and back were debrided, and we observed the following: (1) large areas of skin and soft tissue defects (28 cm × 18 cm) (Fig. 1b), (2) a completely exposed left scapula, (3) many necrotic bones in the scapular ridge, upper pelvic floor and lower pelvic floor, and (4) partially necrotic scapular muscles. The necrotic bone and muscle were then cleared. On the 11th day of admission, the wounds on the left shoulder and back were further debrided. The right lower trapezius myocutaneous flap was overlaid and autologous skin grafting was performed. According to the defect size of the left shoulder and back and the exposure of the left scapula, we designed a 30 cm × 14 cm right lower trapezius myocutaneous flap (Fig. 1c).We cut skin, subcutaneous tissue, and thoracolumbar fascia from the distal part of the flap. We lifted the flap above the shallow layers of latissimus dorsi and rhomboid muscles and then shifted it to the left to cover the defect at the shoulder and scapular ridge. The distal end of the flap was sutured with the skin of the wound after the wound was debrided. We lifted the flap (10 cm × 15 cm in size) on the left latissimus dorsi to cover the scapula of the left shoulder and sutured the fixed flap. The donor site was repaired with medium-thickness skin grafting. The flaps survived completely, and the wound was effectively covered (Fig. 1d). The skin graft on the donor site healed well without complications such as swelling, infection, and necrosis. When the patient was discharged on the 7th day postoperatively, the functions and shape of the shoulder and back were satisfactory.

### Case 2

The patient was a 29-year-old male who was electrocuted by 30,000 V of electricity while working. He was found in a coma, and woke up after 7 min. He was then sent to a local hospital immediately after the injury and transferred to our department for further treatment 6 h afterwards. The patient was very agitated when he entered our department. He had a hoarse voice and no nausea or vomiting. Wounds were located mainly on the left head, neck, back, and left upper limb. The skin surface of the wound was removed, and the basement was pale and could not be touched. There was also evident swelling of the head and neck. The wounds on the neck and back are shown in Fig. 2a. The wound was debrided 4 days after the injury, we observed deep electrical burns on the neck and back, reaching the deep fascia. After we removed the degenerated and necrotic upper trapezius, levator scapulae, and occipital muscles, the size of the skin and soft tissue defect was 28 cm × 10 cm, as shown in Fig. 2b. We designed the right trapezius myocutaneous flap to be about 30 cm × 12 cm in size, as shown in Fig. 2c. We cut the deep fascia deeply from the outside and separated it inwards under the trapezius muscle. Then, we raised the myocutaneous flap from deep inside the lower part and separated it along the nourishing blood vessels. The upper part of the myocutaneous flap was formed from a part of the rhomboid muscle sleeve, and the flap was rotated to the left to cover the wound. The donor site was repaired by medium-thickness skin grafting (Fig. 2d). After the operation, the tissue flap completely covered the wound surface and the electrical burn wound on the neck and back was effectively repaired, as shown in Fig. 2e. The donor site and the flap healed well without any major events. Good cosmetic appearance and function were achieved on the 7th day postoperatively. Half a year after the flap repair, neck and back functions returned to normal and the shape was satisfactory, as shown in Fig. 2f.

## Discussion

Electrical burns are caused by the conversion of electric energy from the body into heat energy causing coagulative necrosis of the skin and deep tissues. Deep tissue damage is often more serious than skin damage. Clinically speaking, it is often characterized by ‘outside of shallowness, deep inside, small mouth, and large bottom’ [5]. High-voltage electrical burns on the head, neck and trunk are common and most of these are caused by direct contact with the power source. They are often accompanied by injuries in deep nerves, blood vessels, muscles, tendons, and bones [6]. Electrical burns in these parts should be paid serious attention to during clinical treatment. Due to the complicated local tissue structure in the head, neck, shoulders, and back, which are adjacent to major organs and large blood vessels [7], if wounds in these areas are not repaired in time, it is easy to damage important organs and cause potentially life-threatening bleeding. Therefore, in the course of clinical treatment, electrical burn wounds should be promptly repaired for the optimal restoration of function and shape.

Previous literature [8], which involved microsurgical anatomy studies and clinical applications of the trapezius myocutaneous flap, have found that the shallow and deep descending branches of the transverse cervical artery have branches that cross the muscles to reach the subcutaneous tissue. These branches line up with the branches of adjacent blood vessels in the superficial fascia and nourish the superficial skin. In addition to nourishing the middle and lower parts of the nutrient trapezius, the vascular network also has a wide agreement with the lumbosacral vessels, providing a reliable anatomical basis for the design of the lower trapezius myocutaneous flap. In-depth studies have found [9, 10] that the trapezius myocutaneous flap has the advantages of reliable blood supply, large tissue coverage, easy operation, and concealment of the donor site. Compared to other commonly used myocutaneous flaps such as latissimus dorsi myocutaneous flaps and pectoralis major myocutaneous flaps, the trapezius myocutaneous flaps are thinner, more flexible, and can provide longer vascular pedicles for dissection. They can be used in reconstructing large areas of tissue defects on the back, maxillofacial, oral cavity, as far as the skull and even the skull base.

According to the anatomical characteristics of the lower trapezius myocutaneous flap and its application in maxillofacial defects, two cases of deep electrical burn wounds on the neck, shoulders, and back were repaired with lower trapezius myocutaneous flaps after timely wound surface debridement. The myocutaneous flaps survived completely and the wounds were effectively repaired without complications such as organ damage and hemorrhage. After the wound was repaired, the function and shape of the electrical burn site recovered satisfactorily, and an excellent clinical curative effect was achieved. This provides a reliable method for the clinical treatment of the deep electrical burn wound on the neck, shoulders, and back. Zhu et al. [11] repaired 12 patients with high-voltage electrical burns in the occipital and nuchal regions. The flaps survived completely in eight cases. In two patients, infection developed in flaps adjacent to wounds with lignification; they healed after the dressing was changed. Necrosis appeared in the distal end of the flap in one case, but healed after re-operation. One patient with surviving flaps died of sepsis and multiple organ failure 21 days after operation. We mainly carried out flap transplantation on the electrical burn wound on the shoulder, neck, and back. The flaps of both patients survived completely without any major events such as infection, liquefaction, necrosis, and the like. Our surgery is similar because we also used lower trapezius myocutaneous flaps to repair the burn wounds. We also tried to avoid cutting too much muscle and ensured that the blood supply of the flap met the needs of wound repair. Therefore, the oedema of the traditional musculocutaneous flap, and defects and dysfunction of the donor site were all avoided.

## Conclusion

In this article, two cases of severe electrical burn wounds on the neck, shoulder, and back were repaired by lower trapezius myocutaneous flaps after the wounds were timely debrided. The myocutaneous flaps survived completely and the wounds were effectively repaired without complications such as organ damage and hemorrhage. The flaps did not have any complications such as infection, necrosis, lignification, swelling, and the like. The donor site was repaired with medium-thickness skin grafting. After the wound was repaired, the function and shape of the electrical burn site recovered satisfactorily, and excellent clinical curative effect was achieved. This study has provided a reliable method for the clinical treatment of deep electrical burn wounds on the neck, shoulder, and back.

## Data Availability

The authors declare that the data supporting the findings of this study are available within the article.
